# Cross-sectional study of the association between functional status and acute kidney injury in geriatric patients

**DOI:** 10.1186/s12882-015-0181-7

**Published:** 2015-11-09

**Authors:** Chia-Ter Chao, Hung-Bin Tsai, Chia-Yi Wu, Nin-Chieh Hsu, Yu-Feng Lin, Jin-Shing Chen, Kuan-Yu Hung

**Affiliations:** Department of Medicine, National Taiwan University Hospital Jin-Shan branch, NO.51, Nan-shih, Jin-shan district New Taipei City, 208 Taiwan; Department of Traumatology, National Taiwan University Hospital, NO.7 Chung-Shan South Road, Taipei, 100 Taiwan; Graduate Institute of Nursing, National Taiwan University, Taipei, Taiwan; Department of Internal Medicine, National Taiwan University Hospital, NO.7 Chung-Shan South Road, Taipei, 100 Taiwan

**Keywords:** Acute kidney injury, Barthel index, Elderly, Geriatrics, Functional status

## Abstract

**Background:**

Patients with chronic kidney disease tend to have impaired functional status, and this can increase the risk of morbidity and mortality. However, no previous studies have rigorously evaluated the relationship between incident acute kidney injury (AKI) and functional status of elderly patients.

**Methods:**

Elderly patients (≥65 years-old) were prospectively from the general medical wards of a single medical center in Taiwan between January, 2014 and August, 2014. These patients were divided into those with and without AKI at initial presentation, according to Kidney Disease Improving Global Outcomes (KDIGO) criteria. Functional status was assessed by Barthel Index on admission. Multiple regression analyses were utilized to investigate the relationship between AKI and functional status.

**Results:**

One hundred and fifty-two elderly patients were recruited, 38.9 % of whom had AKI. Patients with AKI at admission had significantly higher mean Charlson Comorbidity Index score (*p* = 0.05) and borderline lower mean Barthel Index score (34.5 *vs.* 43.1; *p* = 0.08), and a significantly lower bladder continence subscale (5.4 vs. 7.0; *p* = 0.05). Multiple regression analyses indicated that the presence of AKI at admission was associated with a significantly lower Barthel Index score (*p* = 0.04). Increasing AKI severity (higher KDIGO stage) was also associated with significantly lower Barthel Index score (*p* <0.01).

**Conclusions:**

This study documented a close relationship between AKI and functional status in the elderly. Interventions that aim to restore functional status might help to lower the risk of AKI in the elderly.

## Background

Acute kidney injury (AKI) is an emerging public health concern worldwide because it often leads to poor patient outcome [1]. Advanced age increases the susceptibility to acute renal insult and reduces the potential for full recovery because of structural and functional degeneration over time [2]. Aging is also a significant challenge for early diagnosis of AKI in geriatric patients [3, 4]. Consequently, the growth of the elderly population is accompanied by an increase in the incidence of AKI. A U.S. study showed that the incidence of AKI increased progressively with age, from 13.6 per 1000 patient-years in those aged 66–69 years to 46.9 per 1000 patient-years in those aged 85 years and older [5].

Previous cross-sectional and longitudinal studies suggested an association between kidney impairment and risk of functional decline [6, 7]. Thus, individuals with chronic kidney disease (CKD) have 40–70 % higher risk of functional limitation than those without CKD [7]. The mechanism underlying this association has not been fully elucidated, but this relationship may involve traditional factors, such as cardiovascular diseases (hypertension, myocardial ischemia), cerebrovascular diseases, and diabetes mellitus, as well as non-traditional factors such as malnutrition-inflammation syndrome and depression [6, 7].

Depressed renal function in AKI leads to volume dysregulation, electrolyte imbalances, coagulopathy, and increased susceptibility to infections. Moreover, AKI might — similar to CKD — negatively affect the functional status of elderly patients. On the other hand, the functional status of an elderly patient might be a surrogate for comorbidities with prognostic importance because of the complicated interplay between illnesses and an individual’s physical reserve [8, 9]. Besides its influence on prognosis, functional status could theoretically increase the risk for AKI independent of comorbidities and episodes of illnesses, the frequent precipitants of AKI [10]. Most previous studies have treated functional status as a potential outcome-modifier in elderly patients with AKI rather than as a direct determinant of AKI. A Spanish report found that lower Karnofsky Performance Scale (KPS) scores in hospitalized patients with AKI correlated with higher hospital mortality [11] and another cohort study reported that AKI and functional impairment (lower Barthel Index [BI] score) affected mortality in hospitalized nonagenarians [8]. However, few have examined the effect of AKI on functional status or change in functional status.

In the current study, we hypothesize that there is a close relationship between the presence of AKI and the functional status of geriatric patients. We examined this issue by use of a prospectively collected cohort of elderly patients enrolled from general medical wards.

## Methods

### Study design

This study was conducted in accordance with the Declaration of Helsinki. The National Taiwan University Hospital (NTUH) ethics committee approved this study (NO.201306089RINA), and all participants provided verbal informed consent. Family members of elderly patients provided informed consent by use of medical proxy if the patient had dementia, delirium, or disturbed consciousness on admission.

All elderly patients (≥65 years-old) who were admitted to the general medical wards of the NTUH between January 2014 and August 2014 were asked to participate. Age, sex, body mass index (BMI), and history of comorbidities were obtained through chart abstraction and patient interview. The comorbidities were defined as follows: diabetes mellitus (DM) and hypertension (HT) were diagnosed if the medical record had evidence of past or current use of a hypoglycemic medication (DM) and anti-hypertensive agent (HT). Cirrhosis and gastrointestinal (GI) ulcer disease were diagnosed by imaging studies and endoscopic findings. Systolic heart failure was identified by echocardiography (left ventricular ejection fraction <50 %) with compatible symptoms. Coronary artery disease and previous myocardial infarction were defined according to relevant medical records. A history of cancer was confirmed by histopathologic findings, autoimmune diseases were confirmed by serology and clinical diagnoses by rheumatologists, and CKD was defined as an estimated glomerular filtration rate (eGFR) less than 60 mL/min/1.73 m^2^ using CKD-EPI formula. Physical examination parameters, including blood pressure (BP), heart rate, respiratory rate, and consciousness level were also documented at presentation. The attending staff determined the main admission diagnoses of all patients, and divided them into different categories: cardiovascular, pulmonary, hepatobiliary, GI, renal or genitourinary, sepsis of unknown origin, malignancy (treatment or complications), and others. Hemograms were recorded at admission.

### Evaluation of functional status

The functional status of each enrollee was determined based on the BI score. This scale ranges from 0 (totally dependent) to 100 (totally independent) and assays 10 individual aspects of daily living: feeding (0 ~ 10), bathing (0 ~ 5), grooming (0 ~ 5), dressing (0 ~ 10), bowel continence (0 ~ 10), bladder continence (0 ~ 10), use of toilet (0 ~ 10), transfer (from bed-to-chair and return) (0 ~ 15), mobility (on level surfaces) (0 ~ 15), and stair climbing (0 ~ 10) [12]. Dedicated nurse specialists performed the standardized interviews for all patients at admission if possible, and surrogate respondents were used when patients were unable to communicate or unresponsive.

### Evaluation of acute kidney injury (AKI)

AKI was defined according to the Kidney Disease Improving Global Outcomes (KDIGO) guidelines based on the level of serum creatinine (sCr) at admission [13]. In brief, a patient was considered to have AKI if there was a more than 0.3 mg/dL increase in sCr from the baseline value obtained up to 48 h before admission or a 150 % increase in sCr from the value obtained within 7 days before admission. Baseline sCr was ascertained using pre-admission values (within 3 months of admission) or the lowest values within the initial 7 days of admission. The etiology of AKI was classified as predominantly sepsis, shock with hypotension, and others. Sepsis was defined as the presence (probable or documented) of infection accompanied by any systemic manifestations of infection, according to the most recent guidelines [14]. Those with hypotension and shock were categorized as having shock with hypotension-related AKI, irrespective of the presence of sepsis. Admitted elderly patients not fulfilling the KDIGO criteria for AKI were enrolled as the comparative group.

### Statistical analysis

Statistical analyses were performed using SPSS 18.0 software (SPSS Inc., Chicago, IL, USA). We present continuous variables as means ± standard deviations and compared values with an independent samples *t*-test or a Mann–Whitney *U*-test, as appropriate. We present categorical variables as event numbers with percentages, and compared values with a Chi-square test. For evaluation of the incremental effect of AKI on functional status, we selected the functional status at admission (BI score) as the dependent variable, and used multivariate linear regression for analysis, with incorporation of demographics, comorbidities, physical parameters at admission, presence of AKI, diagnosis at admission, and laboratory data. In all analyses, a two-sided *p*-value less than 0.05 was considered statistically significant.

## Results

### Characteristics of enrolled patients

One hundred and fifty-two elderly patients were enrolled during the 8-month study period (Table 1), and the average age was 80.4 ± 8.2 years. More than half of the enrolled patients had comorbid hypertension, 39 % had diabetes mellitus, 22 % had a history of cancer, and 22 % had CKD. A small number of patients had concurrent hemiplegia (3 %) and dementia/Parkinsonism (14 %). The overall mean Charlson comorbidity index was 7.7 ± 2.4. Past reports indicated that elderly Taiwanese patients admitted to an emergency department had a mean Charlson comorbidity index of 3.1 ± 2.1 [15]. A total of 59 patients (38.9 %) presented with AKI at admission. Those with AKI were more likely to have cirrhosis (*p* = 0.03) and higher Charlson index scores (*p* = 0.05). Acute tubular necrosis accounted for 57 (96.6 %) of the cases of AKI, with 39 cases (68 %) predominantly related to sepsis and 10 cases (17.5 %) due to shock with hypotension.

**Table 1:** Baseline demographic and clinical characteristics of elderly patients who presented with or without acute kidney injury (AKI)

Characteristics	Total	AKI (−)	AKI (+)	*p* value
	^……………..^ Mean ± SD or n (%)^a…………….^			
*Demographics*				
Age, years	80.4 ± 8.2	81.0 ± 8.2	79.5 ± 8.3	0.26
Gender, male	77 (51)	42 (46)	35 (59)	0.1
Body mass index, kg/m^2^	22.2 ± 5.2	21.8 ± 6.1	22.7 ± 3.3	0.43
*Comorbidities*				
Diabetes mellitus	59 (39)	31 (33)	28 (47)	0.08
Hypertension	84 (55)	55 (59)	29 (49)	0.23
Cirrhosis	8 (5)	2 (2)	6 (10)	0.03
Obstructive pulmonary diseases	17 (11)	9 (10)	8 (14)	0.46
Coronary artery disease	12 (8)	8 (9)	4 (7)	0.69
Past myocardial infarction	1 (1)	0 (0)	1 (2)	0.21
Heart failure	27 (18)	15 (16)	12 (20)	0.51
Peripheral artery disease	11 (7)	8 (9)	3 (5)	0.42
Chronic kidney disease	34 (22)	18 (19)	16 (27)	0.22
Autoimmune disorder	4 (3)	1 (1)	3 (5)	0.13
Any cancer	40 (26)	21 (23)	19 (32)	0.19
Gastrintestinal ulcer disease	17 (11)	9 (10)	8 (14)	0.46
Presence of hemiplegia	5 (3)	4 (4)	1 (2)	0.38
Dementia and/or Parkinsonism	21 (14)	14 (15)	7 (12)	0.58
*Charlson Comorbidity Index*	7.7 ± 2.4	7.4 ± 2.3	8.2 ± 2.4	0.05
*Main admission category*				0.58
Cardio-vascular	7 (5)	4 (4)	3 (5)	
Pulmonary	69 (45)	46 (49)	23 (39)	
Hepatobiliary	10 (7)	5 (5)	5 (8)	
Gastrointestinal	15 (10)	6 (6)	9 (15)	
Renal	15 (10)	4 (4)	11 (19)	
Sepsis of unknown origin	13 (9)	11 (12)	2 (3)	
Oncology	10 (7)	6 (6)	4 (7)	
Miscellaneous	13 (9)	11 (12)	2 (3)	
*Physical examination parameters*				
Systolic BP, mmHg	134 ± 37.6	143 ± 37.5	120 ± 33.5	<0.01
Diastolic BP, mmHg	74.6 ± 20.1	79.2 ± 21	67.3 ± 16.2	<0.01
Heart rate, beats/min	95.9 ± 21	96.9 ± 19.4	94.4 ± 23.5	0.48
Respiratory rate, breaths/min	20 ± 3.1	20.1 ± 3.1	19.9 ± 3.2	0.7
GCS score	12.5 ± 3.1	12.5 ± 3.2	12.6 ± 2.8	0.98
*Hemogram at admission*				
White blood cell, K/μL	12.2 ± 6.2	11.6 ± 5.1	13.1 ± 7.6	0.15
Hemoglobin, g/dL	11.7 ± 8.5	12.7 ± 10.5	10.1 ± 2.8	0.07
*Baseline sCR, mg/dL*	1.5 ± 1.7	1.5 ± 2.0	1.4 ± 1.0	0.51
*Baseline eGFR* ^b^ *, mL/min/1.73 m* ^*2*^	63.7 ± 30.2	67.1 ± 31.4	58.3 ± 27.6	0.08

^a^Data are expressed as mean ± standard deviation for continuous variables, and number (percentage) for categorical variables

^b^Based on the CKD-EPI formula

*Abbreviations*: *AKI* acute kidney injury, *BP* blood pressure, *eGFR* estimated glomerular fultration rate, *GCS* Glascow Coma Scale, *sCR* serum creatinine

Table 1 also shows the admission data of these elderly patients. Nearly half of the enrollees were admitted for pulmonary disorders, 10 % for GI problems, 10 % for renal disorders, and 9 % for sepsis. The overall mean blood pressure at admission was 134 ± 37.6 (systolic) and 74.6 ± 20.1 (diastolic) mmHg, and patients with initial AKI had significantly lower systolic and diastolic BP (*p* <0.01 for both). The hemogram data and baseline serum creatinine levels were similar in patients with and without AKI, but there was a trend for lower eGFR in those with AKI at admission (*p* = 0.08).

The overall mean duration of hospital stay was 15.8 ± 16.8 days, and patients with AKI had a tendency for longer stays (18.7 ± 22.8 *vs.* 14 ± 11.4 days, *p* = 0.09). Only three patients (2 %), one with AKI and two without AKI (*p* = 0.58), were admitted to the intensive care unit.

### Functional status of enrolled patients

The overall average BI score was 39.8 ± 32.1 at admission, and those with AKI had a tendency for lower scores (34.5 *vs.* 43.1; *p* = 0.08) (Table 2). A previous study reported that the average BI score of healthy community-dwelling elderly in Taiwan was 96.2 ± 8.1 [16]. There were no significant differences in nine of the subscales of the BI, however, patients with AKI at admission had significantly lower scores in bladder performance (5.4 *vs.* 7.0; *p* = 0.048).

**Table 2:** Functional status (Barthel Index score) in elderly pateints with and without acute kidney injury (AKI)

Barthel index	AKI (−)	AKI (+)	*p* value
Total score	43.1 ± 32.6	34.5 ± 30.7	0.08
Feeding	4.9 ± 4.0	4.6 ± 3.8	0.72
Bathing	1.0 ± 2.2	0.7 ± 2.0	0.49
Grooming	1.3 ± 2.2	0.9 ± 2.0	0.29
Dressing	4.2 ± 3.7	3.9 ± 3.6	0.61
Bowel continence	6.9 ± 4.1	6.3 ± 4.3	0.46
Bladder continence	7.0 ± 4.2	5.4 ± 4.4	0.05
Toilet use	4.4 ± 3.7	4.0 ± 3.4	0.5
Transfer (bed-to-chair)	6.3 ± 5.7	5.5 ± 5.3	0.42
Mobility (on level surface)	5.5 ± 5.7	4.5 ± 5.4	0.34
Stair climbing	2.9 ± 3.3	2.0 ± 2.7	0.14

### Relationship of risk for AKI and functional status at admission

We next analyzed the association between AKI and functional status at admission, with accounting for the effect of demographics (age and sex), all comorbidities listed in Table 1 (including CCI scores), admission diagnoses and physical examination parameters (blood pressure and heart rate), and laboratory data (leukocyte counts and hemoglobin level) (Table 3). Multivariate linear regression analysis, with BI score at admission as the dependent variable, showed that AKI at admission (β = −0.15, t = −2.06, *p* = 0.04), comorbid dementia and Parkinsonism (β = −0.31, t = −4.19, *p* <0.01), and higher CCI score (β = −0.18, t = −2.33, *p* = 0.02) were significantly and independently associated with lower BI score. Similarly, another multivariate linear regression model that included the same components as above, but with replacement of the presence or absence of AKI with different KDIGO grades of AKI, indicated that more severe AKI was associated lower BI score among those with AKI at admission (β = −0.2, t = −2.71, *p* <0.01).

**Table 3:** Multiple regression analyses of factors associated with acute kidney injury in elderly adults with Barthel Index score as the dependent variable

Results	*t* value	β coefficient	*p* value
Model 1			
Presence of AKI	−2.06	−0.15	0.04
Model 2			
AKI KDIGO stage	−2.71	−0.20	<0.01

Model 1 included variables from demographic data, comorbidities, and physical examination parameters with the presence or absence of AKI at admission. Model 2 included all the same parameters but with AKI severity (KDIGO stage) at admission

*Abbreviations*: *AKI* acute kidney injury, *KDIGO* kidney disease initiative global outcome

## Discussion

The current study of elderly patients admitted to the general wards of a hospital showed that poor functional status (low BI score) was independently associated with the presence of AKI at admission. The results also showed that among elderly patients with AKI, those with more severe AKI at admission had significantly lower BI scores. This association was independent of the presence of comorbidities and clinical features at admission, suggesting that functional status and the presence of AKI are highly linked in this population. Few previous studies have examined the role of functional status in elderly patients with AKI, and no previous studies have evaluated their potential interaction. Thus, our study, although limited by the relatively small number of cases and a cross-sectional design, could be considered the basis for future studies of this topic. The association we identified provides important insight into the role of functional assessment on the influence of geriatric AKI, an under-appreciated issue.

The mean patient age in our cohort was about 80 year-old, and half of the patients were admitted due to chronic obstructive pulmonary disease (COPD) or pneumonia (Table 1). Previous studies reported that elderly patients with COPD or pneumonia often present with notable functional decline and require hospitalization during episodes of acute exacerbation (AE). One prospective cohort study reported that the average BI scores of nonagenarian in-patients with COPD and AE were 36 ± 23, 50 % lower than the pre-morbid score [17]. Another larger study also found a severe impairment of functional status among those with COPD and AE during hospitalization (BI at admission: 52.5 ± 33.9), and lower scores in those with poor prognosis [18]. Similarly, based on BI scores obtained close to admission and 15 days after admission, Torres et al. found that nursing home residents admitted for pneumonia had lower functional status than community residents (mean BI score: 38.9 *vs.* 85.5), and that those with more impaired activities of daily living (ADL) had significantly greater 1-month and 18-month mortality [19]. Our enrollees had an average BI score of 39.8 on admission, compatible with other studies. Reports that focused on octogenarian in-patients without disease specificity also indicated similar functional impairment during admission [20].

Renal function insufficiency in the elderly is often accompanied by functional deficits. Population-based studies have demonstrated that low eGFR in older adults is associated with 2- to 3-fold higher risk of functional decline and the need for community support, making it difficult for these individuals to live independently [21, 22]. The mechanisms that mediate this relationship may include the presence of chronic inflammation-induced decrease of muscle strength, sarcopenia, or renal osteodystrophy with bony fragility and a tendency toward fracture [23, 24]. Moreover, renal anemia, electrolyte imbalances (i.e. hypokalemia, hyperkalemia, and hyperphosphatemia), and metabolic acidosis could also contribute to the functional decline observed in CKD patients [24]. Some reports indicated an association between CKD and mood disorder or cognitive impairment, and this also may explain the potentiation of functional deficits by CKD in the elderly [25]. On the other hand, patients with CKD and with AKI have several features in common. Both groups have electrolyte imbalances, acidemia, and azotemia, which contribute to appetite loss and malnutrition [26]. In animal models, AKI can upset the balance of pro-inflammatory and anti-inflammatory cytokines, and lead to systemic inflammation [27]. These pathobiologic commonalities might explain the finding that AKI could lead to functional decline in the elderly, which is characterized by failure to undertake self-care.

Another possibility is that lower functional status could be a predisposing factor of AKI in our population. Thus, lower functional status at admission might indicate an overall poorer health status that cannot be explained by the presence of comorbidities. In addition, the lower BI scores in this study could otherwise suggest a greater functional decline from their baseline status, reflecting the severity of their acute diseases. Previous research found that significant declines in functional status among older adults admitted for infection was associated with a more complicated course of hospitalization, during which the risk of AKI increases [28]. Finally, poor functional status could also be a surrogate for clinical instability prior to admission, because it can result from a decreased physiologic reserve from coping with stress, analogous to the concept “frailty”. Previous studies also found that the presence of frailty raised the risk of AKI in individuals with different illnesses [29]. We addressed this possibility by performing an additional logistic regression analysis, with the presence or absence of AKI as the dependent variable. The results indicated that a higher BI score at admission was also negatively associated with AKI at admission (*p* = 0.049). This finding, in combination with our other results, supports the presence of a bi-directional relationship between functional status and AKI in the elderly. However, this relationship must be confirmed by future longitudinal studies, and the strength of this association might differ among patients who have lower or higher scores in the 10 different BI categories.

Our analysis of the 10 different components of the BI indicated that bladder incontinence was significantly more common in patients with AKI (Table 2). Previous large cohort studies also showed that urinary incontinence was significantly associated with functional decline among elderly in-patients [30]. Significant urinary incontinence *per se* could cause a greater functional deficit, but incontinence could also indirectly influence other aspects of functional status, such as mobility [30]. Incontinence in the elderly may indicate an intrinsic genitourinary dysfunction and thereby an increased predisposition to urinary tract infection (UTI), a potential precipitator of AKI [31]. In addition, urinary incontinence is frequently accompanied by bladder catheterization, an event that may precede UTI, and benign prostatic hyperplasia-related incontinence also leads to AKI. Among our cohort, 13 patients (8.6 %) had UTIs as the main diagnosis at admission and those with UTIs had a trend for higher likelihood of AKI at admission (91 % *vs.* 75 %).

Our study has certain strengths and limitations. Through analyzing renal endpoint, we extend the spectrum of potential prognostic influence of functional status to involve AKI among geriatric patients. This topic has not been previously studied. Moreover, we found that the association between functional status and the presence of AKI at admission was independent of baseline CKD status. However, this study is also limited because all patients were from a single center, the design was cross-sectional not prospective, a potentially non-linear effect of BI on outcomes, and the number of cases was rather small. Nonetheless, the importance of our results and their potential generalizability merit further investigation using a larger cohort.

## Conclusion

Studies of the functional status of elderly patients contribute significantly to our understanding of the outcomes of this vulnerable population. In addition to hospitalization course, outcome, and long-term disposition, this study indicates that poor functional status is significantly associated the presence of AKI in the elderly. Moreover, elderly patients with more advanced AKI had significantly poorer functional status than those with less advanced AKI. Further study is needed to confirm and extend our findings by consideration of additional renal endpoints.

## Abbreviations

ADL: Activities of daily living
AE: Acute exacerbation
AKI: Acute kidney injury
BI: Barthel Index
BMI: Body mass index
BP: Blood pressure
CKD: Chronic kidney disease
CI: Confidence interval
COPD: Chronic obstructive pulmonary disease
DM: Diabetes mellitus
eGFR: estimated glomerular filtration rate
GI: Gastrointestinal
HT: Hypertension
KDIGO: Kidney disease injury global outcomes
KPS: Karnofsky performance score
NTUH: National Taiwan University Hospital
OR: Odds ratio
sCr: serum creatinine
UTI: Urinary tract infection
